# Pharyngolaryngeal Involvement by Varicella Zoster Virus: A Rare Case and Literature Review

**DOI:** 10.7759/cureus.91704

**Published:** 2025-09-06

**Authors:** Dzhaner H Bashchobanov, Kristina V Dimitrova, Ivamina A Baltova, Mariya Dimitrova

**Affiliations:** 1 Department of Medicine, Medical University-Sofia, Sofia, BGR; 2 Infectious Disease Clinic, University Hospital "Sofiamed", Sofia, BGR; 3 Clinic of Nervous Diseases, Emergency Hospital "N.I. Pirogov", Sofia, BGR

**Keywords:** cerebrospinal fluid (csf), cranial nerve paralysis, glossopharyngeal nerve involvement, vagus nerve involvement, varicella zoster virus, varicella-zoster virus

## Abstract

Herpes zoster is an acute skin disease that presents after the reactivation of varicella zoster virus (VZV), characterized by clinical manifestations including meningoencephalitis, Guillain-Barré syndrome, vasculopathy, and cranial nerve involvement, such as Ramsay Hunt syndrome, trigeminal neuralgia, and pharyngolaryngeal involvement.

We present the case of a 33-year-old man with unilateral pharyngeal and laryngeal paresis, otalgia, tinnitus, and vesicular rash along the external auditory canal of the left ear. Imaging studies, computed tomography (CT) and magnetic resonance imaging (MRI), revealed no pathology.

The examination of the cerebrospinal fluid (CSF) revealed lymphocytic pleocytosis. Polymerase chain reaction of CSF was positive for VZV DNA. Based on the clinical picture and the results of the CSF examination, pharyngolaryngeal involvement was presumed.

The patient was on combination therapy with antiviral medication and corticosteroid, which showed reversal of neurological symptomatology and reduction of rash.

## Introduction

Varicella zoster virus (VZV, human herpesvirus 3) is a double-stranded DNA virus [1] that belongs to the subfamily *Alphaherpesvirinae*, genus *Varicellovirus*, and leads to the development of chickenpox. After recovery from primary infection, the virus remains latent, usually in the dorsal and trigeminal ganglia, but can also affect any autonomic ganglion, as well as the ganglia of the cranial nerves [2]. While most cases of VZV infection are mild and self-limiting, in some individuals, the virus can lead to severe neurological complications. The most common neurological complications of VZV infection include meningitis, encephalitis, cranial neuralgia, Guillain-Barré syndrome, and arteritis. The cranial nerves most affected by VZV are the trigeminal and facial nerves. Cases of involvement of other cranial nerves have been reported much less frequently [3].

Glossopharyngeal neuralgia, or more precisely vagoglossopharyngeal neuralgia, is a rare pathology that accounts for 1/70 to 1/100 of cranial neuralgias. Its incidence is approximately 0.8/100,000 people [4].

The nervus glossopharyngeus (cranial nerve IX) and nervus vagus (cranial nerve X) are mixed nerves that include motor, sensory, gustatory, and parasympathetic secretory fibers. The motor axons of both cranial nerves originate from a common nucleus, the nucleus ambiguus, and innervate the upper and middle pharyngeal muscles, the muscles of the palatine arch, the soft palate, and the uvula. The sensory fibers of the glossopharyngeal and vagus nerves perform functions of general and specific gustatory sensation. The general sensory fibers of both nerves originate from pseudounipolar cells grouped in two ganglia, the ganglion superior and the ganglion inferior. The dendrites of the pseudounipolar cells form small peripheral nerves and innervate several structures, some of which are the inner ear, the auditory membrane inside, the Eustachian tube, the soft palate, the tonsils, the posterior third of the tongue with the palatine arch and the uvula, the walls of the pharynx, and others. The axons of the sensory cells end in the nucleus tractus solitarius and partly in the nucleus tractus spinalis nervi trigemini [2].

The parasympathetic secretory nerve fibers of the IX cranial nerve innervate the parotid gland, the salivary glands in the buccal mucosa, the tongue, and the pharynx. Together with fibers of the nervus vagus and branches from the sympathetic ganglia cervicale superior, they reach the baroreceptors in the carotid sinus and the chemoreceptors in the glomus caroticus, and participate in the implementation of critical cardiovascular reflexes. The cells of the parasympathetic motoneurons of the X cranial nerve (CN) reach numerous organ plexuses and ganglia at different levels along the course of the sympathetic innervation of the internal organs [2].

The mixed nature of these two nerves and the extensive innervation range show that lesions of the glossopharyngeal and vagus nerves have heterogeneous clinical characteristics. The most frequently described manifestations of neuralgia of the IX and X CN are difficulty in swallowing, taste disturbance on the posterior one-third of the tongue and palate, lack of pharyngeal reflex, parotid gland dysfunction, esophageal motility abnormalities, and autonomic dysfunctions. Oropharyngeal pain is characteristic, provoked mainly by swallowing but also by chewing, coughing, and yawning. The pain is typically neuropathic, episodic, acute, and unilateral, localized in the back of the throat, tonsils, base of the tongue, and at the angle of the lower jaw, lasting from seconds to minutes [5].

Among the most common causes of cranial neuralgia are volume-occupying processes, arteriovenous malformations, and trauma. Infections, such as viruses of the herpes group, *Borrelia burgdorferi*, HIV, *Treponema pallidum*, and several others, are also considered a common etiological cause of cranial nerve damage [6].

Our article aims to present a rare case of a patient with involvement of the IX and X cranial nerves.

## Case presentation

We present a case of a 33-year-old man with complaints of progressive severe pain in the left half of the head for two days. Two days later, speech disorders, difficulty in swallowing, tinnitus, and pain in the left ear appeared. The patient also reported that about a month ago, he had undergone an antibiotic course with levofloxacin due to inflammation of the left ear.

The objective neurological status revealed pseudobulbar syndrome, expressed in moderately severe dysarthria and dysphagia, against the background of the persistent cephalic syndrome covering the left half of the cranium. An indirect laryngoscopy was performed by an otolaryngologist, which verified a fixed left palatine arch and paresis of the left vocal cord. The pharyngeal reflex on the left was absent. In addition, a vesicular-erythematous rash was visualized in the left auditory canal. Erythema of the soft palate and uvula, but without vesicular rash, without pathological changes in the tympanic membrane.

Blood tests did not reveal significant abnormalities. Head imaging was performed (magnetic resonance imaging and computed tomography) without evidence of focal changes in the brain parenchyma and vascular structures. Electromyographic examination also did not reveal any pathology. A lumbar puncture was performed. The general examination of cerebrospinal fluid (CSF) showed mild proteinorachia (0.55 g/L) and lymphocytic pleocytosis. Serum and cerebrospinal fluid were sent for detailed virological analysis. Polymerase chain reaction (PCR) of CSF demonstrated the presence of VZV (Table 1).

**Table 1:** Results from CSF examination VZV: varicella zoster virus; CSF: cerebrospinal fluid

Laboratory indicators	Units	Reference ranges
White blood cells	10.0 x 10^6^	-
Polymorphonuclear cells	1 x 10^6^	-
Mononuclear cells	9 x 10^6^	-
Protein	0.55 g/L	0.15-045 g/L
Glucose	3.94 mmol/L	2.2-4..36 mmol/L
VZV-DNA	Positive	-

Therapy was initiated with acyclovir 5x800 mg (for 7 days), dexamethasone 3x8 mg (for 7 days), mannitol 4x150 ml, and nootropic agents. As a result of the treatment, a reversal of neurological symptoms and a reduction of vesicular erythema in the external auditory canal were observed.

## Discussion

The differential diagnosis of isolated pseudobulbar syndrome is comprehensive. It could include both cerebrovascular diseases, autoimmune diseases, and volume-occupying processes. The etiological causes of the development of pseudobulbar syndrome, although less frequent, also include infectious diseases. In the case presented above, despite the absence of toxic-infectious syndrome, the presence of cephalic syndrome and vesicular-erythematous rash is indicative of the search for a viral etiology of the disease. Most often, the involvement of cranial nerves in infectious diseases is included in the spectrum of basal meningoencephalitis, which is caused by infections with Mycobacterium tuberculosis, HIV, Treponema pallidum, and others. Isolated cranial neuritis, which is complicated by viral infections, is much less common. The herpes group of viruses, which includes the varicella-zoster virus, is an example of such an infection. However, cranial nerve IX and X involvement is a rare manifestation of the disease [6].

A retrospective study by Tsau et al. included a total of 330 patients with VZV-induced cranial neuritis [7]. Their report reported only three patients with isolated glossopharyngeal nerve involvement (0.9%) and three with vagus nerve involvement (0.9%). The results of the study also indicated that lesions of more than one cranial nerve are rare. They described only three patients with simultaneous involvement of cranial nerves IX and X. Still, in all three cases, it was in combination with lesions of the remaining posterior fossa cranial nerves (cranial nerves VII and VIII).

A few years earlier, Nisa et al. published a review article based on data from a total of 65 studies on cranial neuritis due to viral infection [8]. Fifty-four of the described cases are of pharyngolaryngeal involvement caused by VZV. Seventeen percent of these cases have isolated involvement of only IX and X cranial nerves. One-third of all patients had otalgia, and in two-thirds, a vesicular rash was present. An interesting fact, according to the report, is that 60% of the cases of otalgia were left-sided. MRI results were expected in 69% of the described cases, and CSF examination showed lymphocytic pleocytosis in 55% of cases [8].

The clinical case presented by us corresponds to the data published so far in the world literature. Although rare, the varicella-zoster virus can lead to neuritis of IX and X, the manifestation of which is a cephalic syndrome, otitis, and paralysis of the soft palate, uvula, and vocal cords. Due to the involvement of the peripheral part of the cranial nerves, quite logically, no focal pathology was detected in brain imaging studies. Lumbar puncture and cerebrospinal fluid examination revealed lymphocytic pleocytosis and mild proteinorachia, consistent with the viral nature of the disease. PCR showed the presence of viral particles.

Due to the low incidence of the disease in the general population, both diagnosis and subsequent treatment pose significant challenges. In their literature review, Nisa et al. noted that 57% of patients with pharyngolaryngeal involvement received corticosteroid therapy, and in 1/3 of them, glucocorticosteroids (GCS) were combined with an antiviral agent [8]. However, full recovery was observed in only 26% of all described cases. In the clinical case we described, we used a combination of antiviral agents, corticosteroids, anti-edema, and nootropic therapy. The daily dosage of the medications was the recommended dose per kilogram of body weight (acyclovir 10 mg/kg IV q8h). Against the background of the treatment, we observed a complete reversal of the neurological deficit.

## Conclusions

Neuralgia of the IX and X CN is a rare complication of VZV. It should always be suspected in the presence of dysphagia, dysarthria, otalgia, cephalalgia, and especially a vesicular rash in the external auditory canal. Timely diagnosis and initiation of treatment with antivirals, corticosteroids, and anti-edema agents give good results and, in some cases, complete resolution of the clinical picture.
